# Brain Metastasis With a Solitary Lesion Secondary to Knee Joint Ewing Sarcoma: A Case Report

**DOI:** 10.7759/cureus.39612

**Published:** 2023-05-28

**Authors:** Muhammad Irfan, Osama Abdelsamad, Han Grezenko, Anshum Patel, Muhammad Rizwan Akram

**Affiliations:** 1 Department of Neurosurgery, Nishtar Medical University, Multan, PAK; 2 Department of Neuroscience, Sheikh Zayed Hospital, Rahim Yar Khan, PAK; 3 Department of Clinical Oncology, Khartoum Oncology Hospital, Khartoum, SDN; 4 Department of Research, Michigan State University, Michigan, USA; 5 Department of Neuroscience, Barrow Neurological Institute, Phoenix, USA; 6 Department of Internal Medicine, Narendra Modi Medical College, Ahmedabad, IND; 7 Department of Internal Medicine, Sheikh Zayed Medical College, Rahim Yar Khan, PAK; 8 Department of Internal Medicine, BronxCare Health System, New York, USA

**Keywords:** rare incidence, aggressive malignancy, brain metastasis, solitary lesion, outcome, ewing sarcoma

## Abstract

Brain metastasis from Ewing sarcoma is rare and can present with various symptoms. We present a 21-year-old female who underwent surgery for Ewing sarcoma of the knee joint and, after six months, was reported with complaints of headache and vomiting. Considering recommended investigations, metastatic Ewing sarcoma of the brain was diagnosed, and a treatment protocol, such as a combination of surgery, chemotherapy, and radiation, was given. Our observation shows this is the first case reported with a solitary metastatic brain lesion associated with Ewing sarcoma.

## Introduction

Ewing sarcoma is an aggressive malignancy that affects bones and soft tissue, with a higher incidence in young adults and children. It is usually found in the diaphysis of long bones of the arms and legs but can also occur in the spine, pelvis, chest wall, ribs, and skull. It usually spreads to the lungs and other bones and rarely metastasizes to the brain. Metastatic brain tumor associated with Ewing sarcoma is rare. A few cases have been studied in the existing literature [1]. The most common symptom of metastatic brain Ewing sarcoma is a headache accompanied by nausea, vomiting, and changes in vision. Other symptoms may include confusion, seizures, and problems with coordination and balance. A combination of surgery, chemotherapy, and radiation is paramount for treatment. Surgery removes the tumor from the brain, while radiation therapy targets and destroys any remaining cancer cells. Chemotherapy helps to shrink the tumor and stop the spread of cells. Our case discussed here is also associated with a single metastatic lesion in the right frontal region secondary to Ewing sarcoma of the knee joint. After surgery for a tumor, the patient was discharged, followed by radiation therapy sessions at regular intervals. This case report describes a rare case of brain metastasis with a single lesion from Ewing sarcoma and highlights its unique finding.

## Case presentation

**Figure 1:**
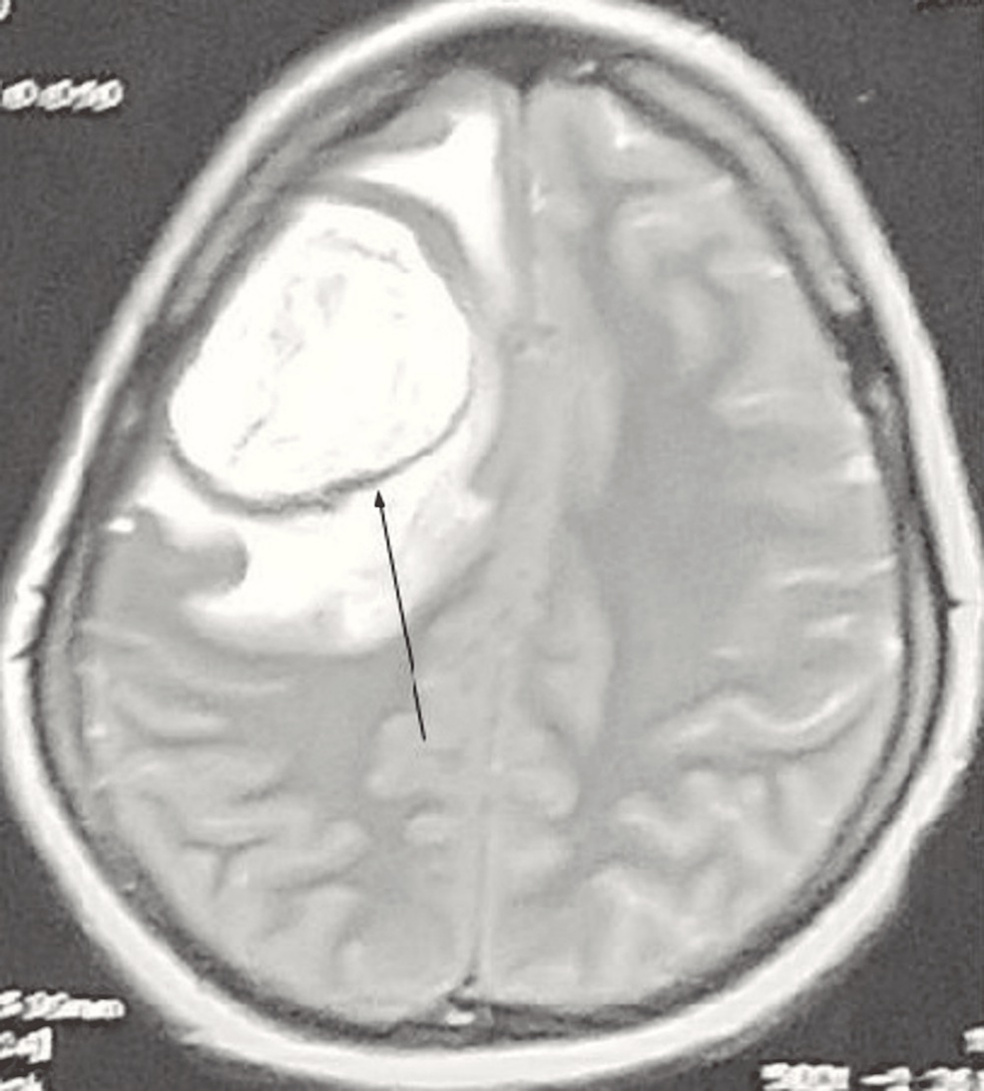
Axial MRI of the brain (T2-weighted sequence) showing enhancing hyperdense mass lesion in the right frontal lobe (arrow).

**Figure 2:**
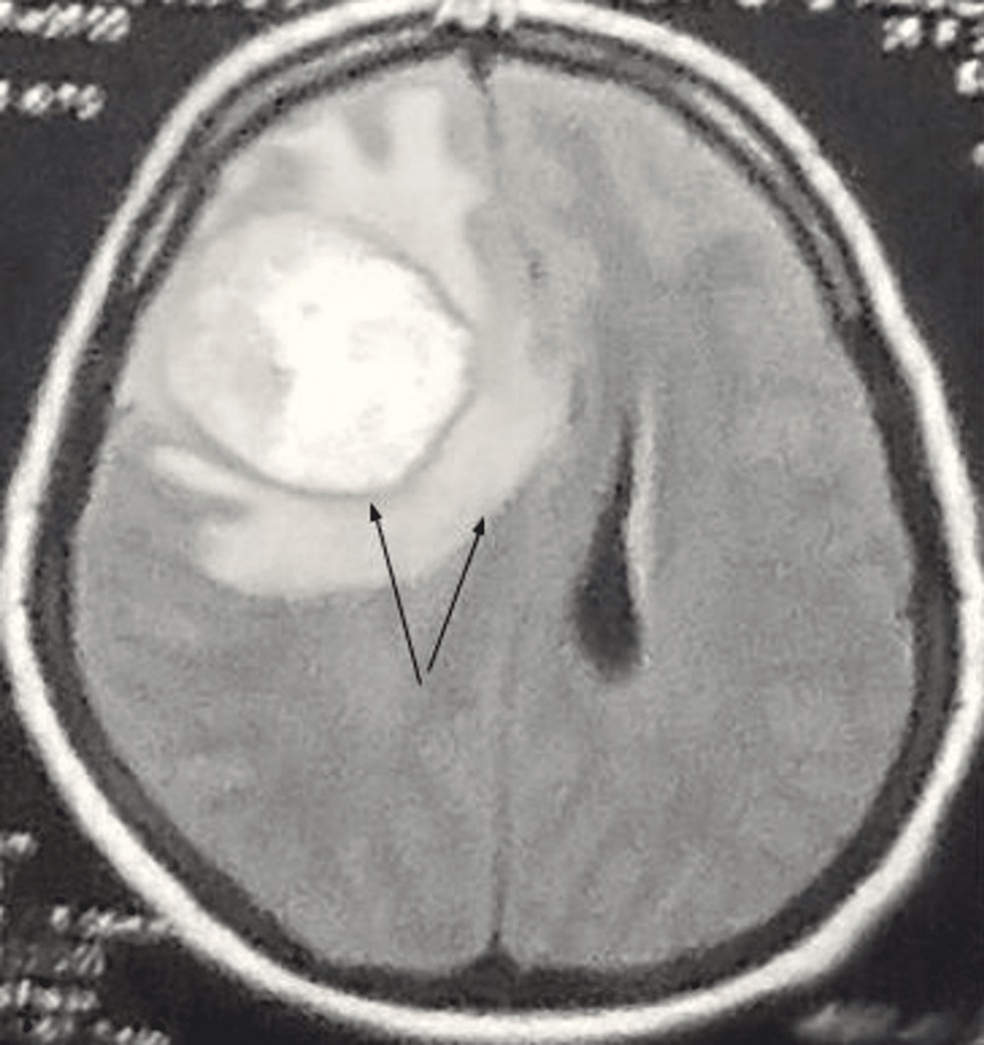
Axial MRI of the brain (FLAIR-weighted sequence) demonstrating lesion in the right frontal lobe with perilesional edema (arrow). The mass effect of the lesion causes a midline shift of 15mm towards the left side. FLAIR: fluid-attenuated inversion recovery

We present a 21-year-old female who complained of difficulty walking and swelling on the medial side left knee for two months. Her left knee joint examination revealed a limited range of motion. On investigation, the hematological profile, renal function test, and liver function test were within normal limits. The abdominal ultrasound report, however, showed two inguinal lymph nodes. The X-ray and magnetic resonance imaging report revealed well-defined lobulated approximately measuring 7 x 10 cm mixed intensity mass in the medial compartment of the left distal thigh with mild perilesional edema, while tissue biopsy from the left knee joint showed grossly necrotic and hemorrhagic areas and histologically malignant small round blue cells with the possibility of Ewing sarcoma of the left knee joint. The diagnosis was further confirmed by immunohistochemistry. The tumor was positive for cluster of differentiation 99 (CD99) and transducer-like enhancer of split 1 (TLE1). The post-surgery patient commenced chemotherapy with six cycles of the VAC regime (vincristine sulfate, dactinomycin, and cyclophosphamide) and 30 sessions of Cobalt-60 external beam radiation therapy and was kept on follow-up. After six months, the patient was hospitalized with complaints of severe headaches and multiple episodes of vomiting for one month. On examination, she was alert and conscious. Her vital signs were within normal limits. Her neurological examination exhibited left-sided weakness; however, cranial and sensory findings were normal. Her vital signs showed that her temperature was 98.8 F, pulse rate was 80 per minute, respiratory rate was 16 per minute, blood pressure was 110/90, and body mass index was 18. While her pupils were reactive to light, Babinski's sign was positive, and her Glasgow Coma Scale (GCS) score was 15/15. However, there were no findings on other systematic examinations. Brain magnetic resonance imaging with intravenous Gadolinium contrast in axial view showed ring enhancing hyperdense mass lesion in the right frontal lobe associated with significant perilesional edema causing mass effect on adjacent brain parenchyma, and frontal horn and body horn of ipsilateral lateral ventricle, and midline shift (Figures 1, 2). Images were taken in weighted sequences of T1, T2, and fluid-attenuated inversion recovery (FLAIR).

**Figure 3:**
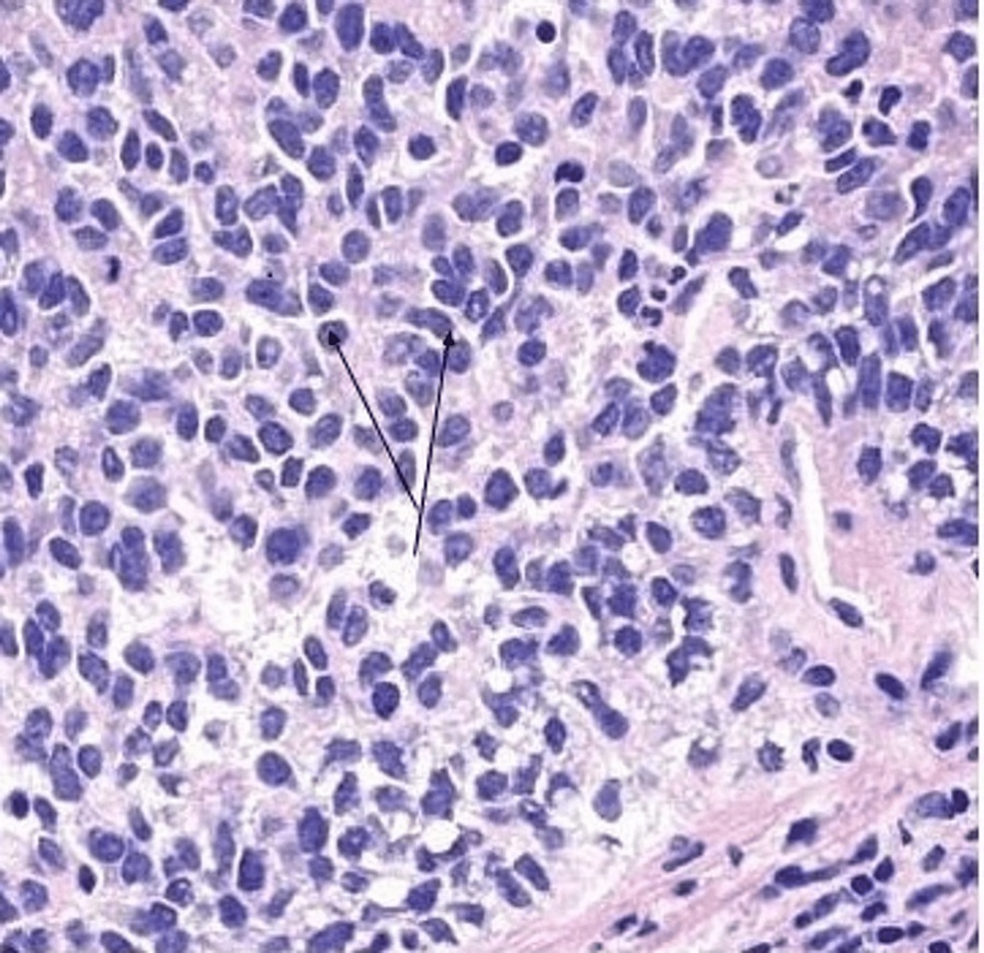
The tumor cells show small round cell appearance with focal pleomorphism.

**Figure 4:**
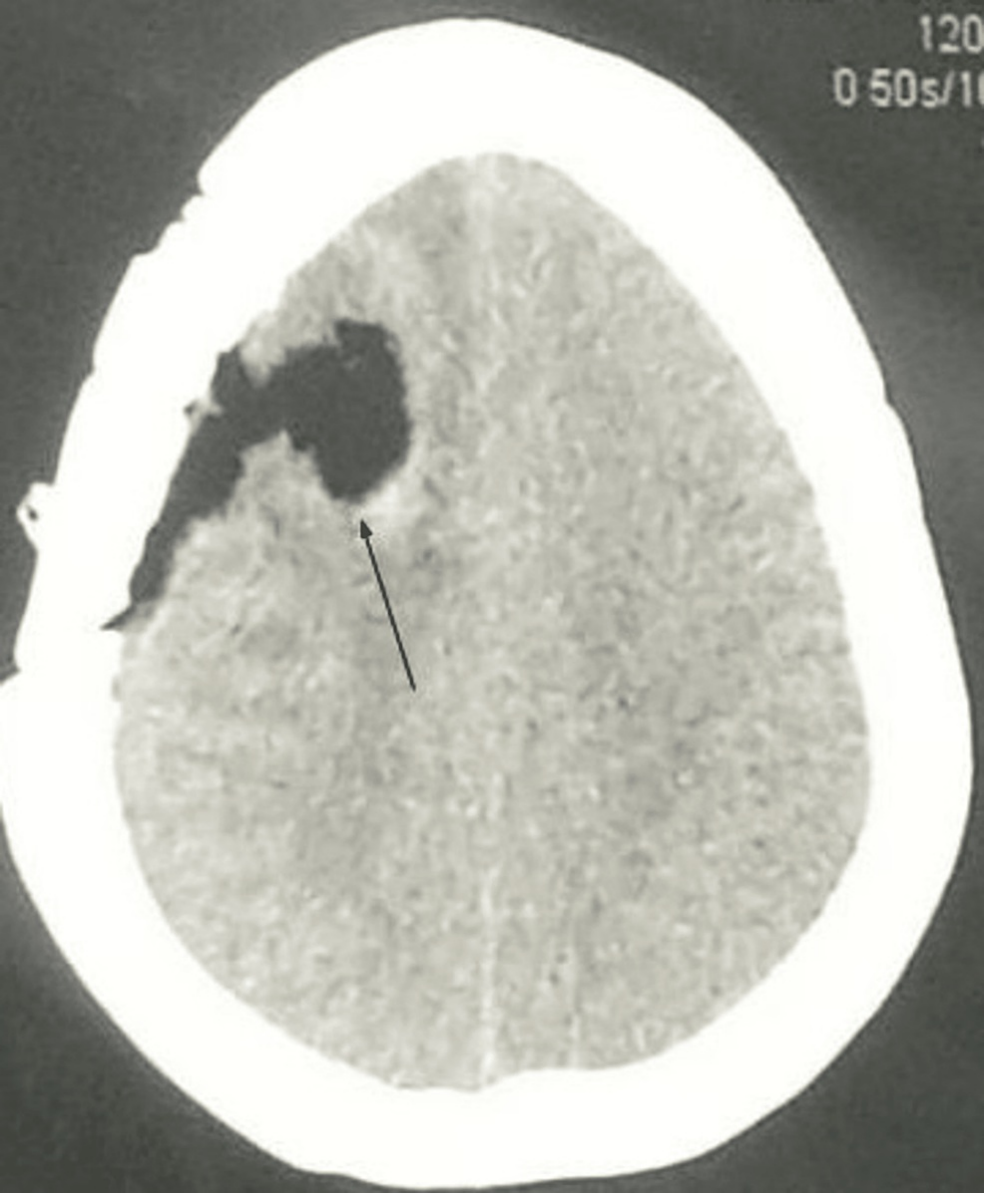
Post-operative CT scan shows excision of brain tumor (arrow)

The serum blood test showed a red blood cell count of 5.571 x 10^6/ul, a hemoglobin level of 11.9g/dl, a mean corpuscular volume of 85.6fl, hematocrit 35.5%, platelets 228 x 10^3/ul, white blood cell counts 14.20 x 10^3/ul, neutrophils 80% and lymphocytes 18%. Random serum glucose was 95 mg/dL. In comparison, liver function tests, serum electrolytes, and coagulation reports were within normal limits. After undergoing extensive investigations for surgery, being cleared by an anesthesiologist, and taking informed consent, she was prepared for the surgical excision of a brain tumor. Highly vascular tissue mass was excised from the right frontoparietal region with a modified uni-coronal incision via an open skull surgical approach, and a biopsy sample was carried out for histopathology, which, later on, came out to be positive for immunohistochemistry markers such as CD99 and TLE1 with a concurrent diagnosis of metastatic Ewing sarcoma of the brain (Figure 3). After the surgical procedure, the patient was observed in the recovery room for 24 hours, and the neurological examination was intact. A post-operative CT scan was done the next day as shown in Figure 4. After one week, she was discharged from the neurosurgery ward and kept at follow-up with 30 Gy of whole brain radiation therapy and 15 Gy narrow field irradiation sessions to the right frontal lobe at regular intervals.

## Discussion

Ewing's sarcoma is a rare malignant bone tumor most commonly affecting children, adolescents, and young adults. It is, however, the most common childhood tumor to cause secondary brain metastasis. In terms of incidence, it is the second most common malignant childhood bone tumor, with an average age at diagnosis of 15 years old. American Cancer Society showed that Ewing sarcoma accounts for only about 1% of all pediatric cancers. Most cases occur in Caucasian individuals, with a higher incidence in males than females. Ewing sarcoma cases have been reported more in the United States than in other countries. Each year 200-250 new cases are diagnosed in the United States. It is found in the long bones of the arms and legs, but it could be seen in the pelvis and ribs with an incidence of 10 to 20% and less than 5% in the skull bones.

The exact cause of Ewing sarcoma is unknown, but it is believed to be the consequence of genetic mutation. This mutation is thought to occur in a gene that regulates cell growth and division, leading to an uncontrolled proliferation of cancerous cells. These changes can be inherited or acquired through environmental factors. Exposure to radiation and certain chemicals has been associated with a high risk of developing the disease. The study has shown that chromosomal translocation t(11, 22) (q24; q12) is found in more than 90% of Ewing sarcomas, resulting in the formation of the EWSR1-FLI1 fusion gene (Ewing sarcoma breakpoint region1-Friend Leukemia integration 1 transcription factor), and its detection helps in confirming the diagnosis [2].

Ewing sarcoma spreads to other body parts, such as the lungs, bones, and lymph nodes. Ewing sarcoma is most likely prone to metastasis from its primary site due to several risk factors, such as larger tumor size, advanced age, specific genetic mutations, certain treatments such as radiation therapy, and specific locations of the body such as the chest or abdomen. However, metastases to the central nervous system have most recently been estimated to occur in less than 5% of cases [3]. They are usually due to the direct extension of a bony lesion into the extradural space or, more rarely, through hematogenous spread [4]. The existing literature reported few cases of central nervous system extraosseous Ewing's sarcoma (CNS-EES) [5]. In this study, the tumor involved the skull bone along with the overlying temporalis muscle in addition to cerebral parenchyma. Metastatic lesions of Ewing sarcoma in the intracranial compartment could be misdiagnosed as central primitive neuroectodermal tumor (c-PNET), such as medulloblastoma, because of the similarity in their histological appearance. Therefore, diagnosis is confirmed based on immunohistochemistry, cytogenetics, and histopathological examination. A tumor could metastasize to the dura mater membrane of the brain. Dural mater membrane metastasis accounts for 9% of all CNS metastasis, as described in a study [4]. This study also showed that lesions could involve the skull with diffuse, massive epidural and subdural plaque-like nodules. Our case discussed here is a metastatic Ewing sarcoma to the brain from the knee joint with complaints of headache and vomiting. Previous research revealed a few cases of metastatic Ewing sarcoma to the brain parenchyma, which is less than 4.3%, as shown in a case report [1]. It is also seen in earlier literature that brain metastasis lesions are usually seen in multiple patterns. Our case has another significance due to its presentation as a single lesion.

Treatment of metastatic Ewing sarcoma of the brain depends on several factors. The treatment options available for patients with CNS-EES are similar to those for EES elsewhere in the body. For localized Ewing sarcoma, multiagent chemotherapy regimens, including cyclophosphamide, ifosfamide, doxorubicin, dactinomycin, and etoposide, have been proven to be effective. However, multimodality treatment, including surgery, chemotherapy, and radiation, is utilized for metastatic tumors [5]. The diagnosis of Ewing sarcoma is confirmed by testing for the EWSR1 mutation that excludes other types of embryonal CNS tumors. Long-term disease-free survival is possible with adherence to the appropriate therapeutic regimen after gross surgical resection [6].

The treatment plan for metastatic brain Ewing sarcoma includes surgery followed by chemotherapy and radiation. Also, it involves collaborating with a multidisciplinary team of specialists to determine the best course of action according to the patient's needs. As in our case, a surgical treatment plan via a modified coronal approach was utilized after a common consensus among a multidisciplinary team of specialists. This surgical approach is well suited for the tumor located in the frontal and parietal lobes of the brain. Other surgical treatment approaches, such as craniotomy and endoscopic and stereotactic surgery, could be considered for metastatic brain tumors [7]. These surgical approaches are utilized depending on various factors, such as the location and size of the tumor, the number of tumors present, and the patient's overall health. The prognosis of Ewing sarcoma depends on its site and size, as well as the patient's age and overall health. The prognosis of Ewing sarcoma is generally poor for patients with metastatic disease, especially in the CNS, with a five-year survival rate of less than 30% [8]. However, this aggressive neoplasm is curable, with the best prognosis observed in patients younger than 16.

This case report is crucial for neurosurgeons and oncologists in terms of the fact that metastatic Ewing sarcoma of the brain should be taken into account as one of the differential diagnoses of a brain tumor with its varying presentation from single to multiple lesions so that earlier identification of the tumor based on its specific confirmatory investigation such as immunohistochemistry and histopathological examination can be made. The required treatment modality could be utilized. In addition, it is also encouraged that more research on this rare metastatic brain tumor is needed for a better understanding of these tumors in terms of diagnosis and treatment protocols.

## Conclusions

The presence of a metastatic brain tumor secondary to a knee joint Ewing sarcoma is concerning and requires prompt medical attention. Our case report highlights the significance of considering Ewing sarcoma as a potential diagnosis in patients with a history of bone cancer who present with neurological symptoms. Early diagnosis and prompt treatment are critical for improving outcomes in this rare and aggressive malignancy. 
